# Measuring the strength of primary care: development of a new system of Structural Indicators for the Strength of Primary Care – SiSPC

**DOI:** 10.1017/S1463423625100509

**Published:** 2025-09-30

**Authors:** Wienke G.W. Boerma, Peter Groenewegen, Rob Timans, Sarah Burgmann, Rosa Suñol, Pili Illarramendi Charovsky, Jose M. Valderas

**Affiliations:** 1 Netherlands Institute for Health Services Research (Nivel), Utrecht, Netherlands; 2 Austrian National Public Health Institute, Vienna, Austria; 3 Fundació Avedis Donabedian, Barcelona, Spain; 4 Centre for Research in Health System Performance (CRiHSP), Yong Loo Lin School of Medicine, National University Health System, Singapore

**Keywords:** indicators, international comparison, primary care

## Abstract

**Aim:**

The aim of this study was to develop an up-to-date system of Structural Indicators for the Strength of Primary Care (SiSPC) to enable comparisons of primary care systems across countries.

**Background:**

Indicators are needed for international research into the development of primary care and to support countries in monitoring improvements in access, responsiveness and efficiency of their primary care services. International comparisons with use of identical indicators for the strength of primary care offer policymakers opportunities to learn lessons from abroad.

**Methods:**

Our point of departure was the Primary Health Care Activity Monitor Europe (PHAMEU), that effectively measured the strength of primary care at the beginning of this century. We went through the following steps: (1) Reduction, refining and tuning of the PHAMEU indicator system (2) comparison with the European Primary Health Care, Impact, Performance and Capacity Tool (PHC-IMPACT) (3) addition of topics from other frameworks (4) identification of topical issues from the literature. The resulting draft indicator system was discussed at meetings and received feedback from experts from 25 countries.

**Findings:**

SiSPC consists of three care-related domains: Structure of Primary Care, Systemic Aspects of Facility Management and Systemic Aspects of Care Delivery. SiSPC also contains a domain on the Context of Primary Care. Care processes that vary between care providers, were not included as a domain at the system level.

The health needs of the population have strongly changed over the years (Mackenbach, 2019). Demographic changes have led to a growing number of older people, often living in the community. These changes, coupled with changes in life-style, have resulted in rising numbers of people with a chronic disease, especially those with several chronic diseases. (Adan *et al.*, 2020). Health care systems struggle to find an answer to the quantitative and qualitative changes in healthcare needs that result from these changes (Rijken *et al.*, 2018). Patients with (multiple) chronic conditions need accessible healthcare services with a focus on their person, and taking their context and medical history into account, rather than on their disease(s). Instead, today’s health care systems are usually fragmented with specialist care provided in mono-professional silos and with a dominant single-disease orientation. Furthermore, possible connections between health needs and social needs are easily overlooked (Akiya *et al.*, 2021).

Primary care indicates the part of the health care system that delivers ambulatory, general medicine first-contact care to the community-dwelling population. The term primary health care is more action-related and refers to a policy approach to achieve universal health coverage and equal access to basic and affordable health care services for everybody. We focus on primary care as service delivery. Many countries aim to strengthen primary care as a strategy to make their health care systems more person-centred. Person-centeredness is a key characteristic of primary care, in addition to a generalist and community-oriented approach. Strong primary care provides accessible and continuous care of a comprehensive nature; if patients are in need of this, care provided by specialists or mental and social services is coordinated (Starfield, 1994).

Previous international comparative research has shown that countries differ in the strength of their primary care systems. Such differences are related to aspects of quality and costs of care (Kringos *et al.*, 2013a). International comparative studies will continue to be needed to provide policymakers with guidance to tune their health care systems, including primary care, in line with policy goals. A comprehensive, integrated and well-founded system of indicators for the strength of primary care must be part of such comparisons.

The reason for the development of our system of Structural Indicators for the Strength of Primary Care (SiSPC) was linked to the PaRIS project. This is the International Survey on Outcomes and Experiences of People Living with Chronic Conditions, initiated by the Organisation for Economic Cooperation and Development (OECD). The PaRIS project was coordinated by a consortium, led by the Netherlands Institute for Health Services Research (Nivel) (OECD, 2025). We developed SiSPC parallel to this project to enable answering future research questions related to health system and country-level influences on patient-reported experiences and outcomes. In our approach of developing the SiSPC indicator system we started with the PHAMEU (Primary care activity monitor Europe) framework. With the PHAMEU indicators the strength of primary care systems has been measured in 31 countries (Kringos, 2012). These indicators have also later been used in the international QUALICOPC study (Schäfer *et al.*, 2019) as well as other studies (Hansen *et al.*, 2015) and have been at the basis of policy initiatives in individual countries. Although the starting point for the development of SiSPC was the OECD PaRIS project, the focus will be broader. We also include the (European) countries where the PHAMEU indicator set was measured and that are not OECD members (Bulgaria, Cyprus, Malta, North Macedonia), non-OECD members that participated in PaRIS (Romania and Saudi Arabia), and the non-European countries that participated in the QUALICOPC project (Australia, New Zealand, Canada).

We had the following arguments for developing an updated framework and system of indicators. The PHAMEU indicators reflect the situation at the beginning of this century. Monitoring today requires more recent developments in primary care to be taken into account. Changes in primary care and in health needs in populations, societal priorities and other conditions, not only call for an update, but probably for an extension as well. A new and up-to-date dataset will allow for current comparisons of countries. The research presented here aimed to develop an up-to-date system of indicators to be used for the measurement of the strength of primary care.

## Methods

The indicator system to be developed should satisfy a number of requirements:

- Focus on the national system level: indicators should exclusively focus on the way primary care is structured, organized and regulated at country or system level.
- Topical: new insights and developments on the role of primary care in the context of health care systems should be represented.
- Balanced orientation: the focus of the PaRIS project is on service provision to patients living with chronic diseases. However, SiSPC aims to have a balanced approach and should also reflect primary care’s broad attention to prevention and treatment, acute and chronic disease, palliative care, and to people of all ages.
- System diverse: indicators must be relevant and applicable to various health care systems (e.g. in higher-middle- and high-income countries in Europe and OECD member states outside Europe).
- Evidence-based: indicators should be supported by evidence in the international literature for the relationship with outcomes, be they survey-based patients’ experiences and outcomes, or other, aggregate outcomes at country level.
- Comparable and valid: information to be collected must be recent, valid and as comparable as possible.
- Feasible and parsimonious: indicator items should be measurable, preferably with easily accessible sources of information and they should have added value.

To develop SiSPC, we have gone through the following steps.

1. Reduction of the number of PHAMEU indicators. The core development team (WB and PG) reviewed the PHAMEU indicator items critically on their suitability for SiSPC. We also analyzed the contribution of individual items to the latent variables that formed the dimensions of PHAMEU and deleted those with low item-total correlations. What remained after this step served as the groundwork for the development of SISPC.
2. Merging the remaining items of PHAMEU and the WHO PHC-IMPACT Tool. The recent ‘European Primary Health Care, Impact, Performance and Capacity Tool’ (PHC-IMPACT) is a comprehensive system of indicators that fits very well with the structure of PHAMEU (WHO, 2019). Therefore, we decided to merge it with what remained of PHAMEU after step 1 to have a firm body of indicators before searching and reviewing other sources. PHC-IMPACT is a service tool for WHO Member States. Key informants and surveys are frequently mentioned as sources. Until now, we have seen no database with measurements of the indicators from PHC-IMPACT. PHAMEU provided measurement of all indicators and this is also the aspiration of SiSPC.
3. Adding relevant elements from other frameworks. Our search for frameworks aimed to compensate for possible limitations or omissions of PHAMEU and PHC-IMPACT. We scanned the literature to identify other relevant frameworks. The scan was done in the context of developing the overall conceptual framework of the PaRIS project; we refer to the Methods section in Valderas *et al.* (Valderas *et al.*, 2024, De Boer *et al.*, 2022). A separate search was made for Spanish-speaking countries in the Americas to capture the specific approach provided in those countries. We restricted the search to national and international primary care frameworks from 2010 and later (previous ones having been incorporated in PHAMEU).
4. Integrating topical issues. We view topical issues both as current challenges in primary care and key elements of a vision of what is needed for primary care to cope with these challenges. We identified topical issues through a review of reports, documents and policy briefs from professional and international organizations, available on the internet and published after 2010. The selected publications were independently examined by two researchers to identify insights, visions and innovations that should make primary care fit for the future. Agreed topics have been listed and examined again in the light of the requirements. For each of the resulting topics, it was decided whether they were covered by the results of the previous steps. If not, indicators were formulated by two researchers in a consensus procedure.
5. Processing internal and external feedback on the development process and its result. At several occasions in different stages of the process the core development team (WB and PG) received feedback. Members the consortium that implemented the PaRIS surveys provided feedback on provisional versions of SiSPC at team meetings in 2022, 2023 and 2024. In September 2022, the provisional framework was presented and discussed at a conference of the European Forum for Primary Care (EFPC) in Ghent (Belgium). As a final test, the draft version of SiSPC was distributed in December 2024 among primary care experts in 34 countries, including the National Project Managers (NPMs) of the PaRIS project. As these experts will be involved in the first data collection round of SiSPC, they were particularly invited to focus on the relevance and clarity of the indicator items and the workload and feasibility of data collection. These various occasions of review and feedback aimed to identify and repair redundancies, gaps, poor validity and measurability. A recent overview of the evidence for aspects of strong primary care was provided in an EFPC Position Paper (Akman *et al.*, 2022). However, in particular for emerging topics the availability of evidence is likely to lag behind. In those cases, inclusion was based on consensus in the research team.

## Results

**Figure 1.**
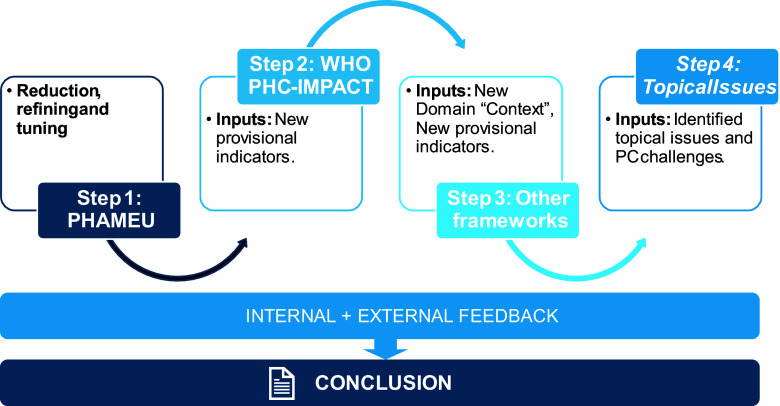
Overview of steps in the development of SiSPC.

The results will be presented in line with the steps mentioned in the previous section. This has been visualized in the flow chart of Figure 1.

### Step 1: Review and tuning of the PHAMEU framework and indicators

**Figure 2.**
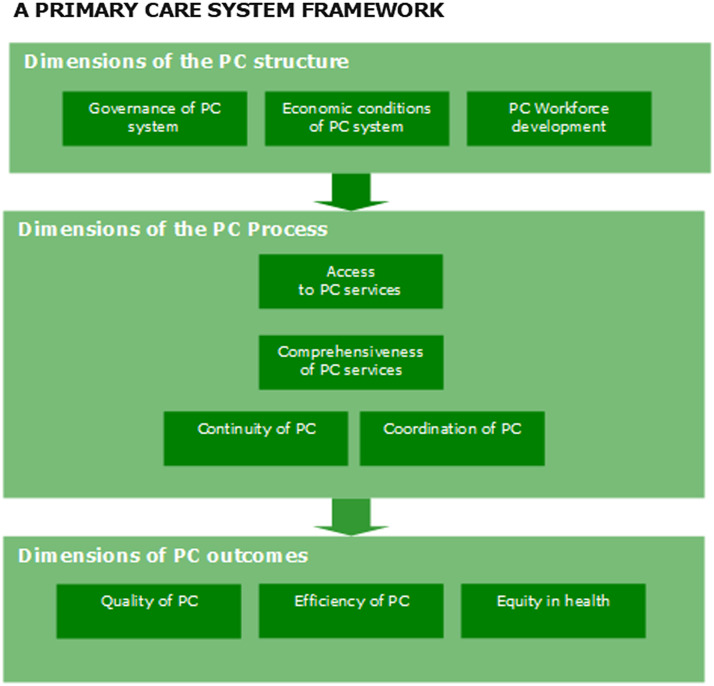
The PHAMEU framework of primary care.

Following Donabedian, the PHAMEU framework distinguishes the broad domains structure, process and outcomes, which are broken down to dimensions, as depicted in Figure 2 (Donabedian, 1988, Kringos *et al.*, 2010, EXPH, 2017).

We reviewed the framework in line with our requirements, resulting in the following decisions. The three dimensions of Domain 1 Structure of primary care (Governance; Economic conditions of primary care; Primary care Workforce Development) have been maintained.

Concerning Domain 2 (Process of primary care) we made a distinction between indicators that should be measured at system level and others that can more suitably be measured through surveys among providers and patients. Our main criterion was whether process indicators vary between care providers within countries or not. Only if they do not (substantially) vary, we have adopted them as system characteristics. The application of this criterion led to the removal of 24 indicators. Those we maintained were labelled ‘Systemic aspects of care delivery’. We use the term ‘systemic’ to emphasize that these indicators are measured at (national) health system level and not at the level of primary care practices.

Domain 3 (Outcomes of primary care) was excluded altogether as it does not measure the strength of primary care but the consequences of strong primary care. This led to the removal of 30 indicators on outcomes.

We identified a gap in the framework related to Domain 1 (Structure of primary care). We added indicators, covering national regulation that facilitate service delivery and local management. Such regulation can provide important incentives to organize the delivery of care professionally, e.g. by stimulating local practice management. Since the conceptualization of PHAMEU, regulatory aspects of primary care delivery have changed. We therefore included this new domain, and labelled it ‘Systemic aspects of facility management’.

In addition to the critical review of the framework, the PHAMEU indicators have been analyzed statistically to assess their contribution to the overall dimensions by computing item-total correlations. In previous studies, separate indicator items have rarely been used to characterize primary care systems. Usually, they have been combined into the dimensions through latent variable analysis. We have excluded 23 indicators with low correlations to the total score for each dimension. An example of an indicator, removed based on this analysis, is one about whether primary care has a separate department or unit within the Ministry of Health. This indicator had a low correlation to the overall dimension of Governance. The review of the PHAMEU framework (as described above) and the statistical analysis on the indicators’ contribution to the latent variables altogether resulted in exclusion of 77 from the original 122 indicators of PHAMEU.

### Step 2: Merging the remaining indicators of PHAMEU with WHO PHC-IMPACT

**Figure 3.**
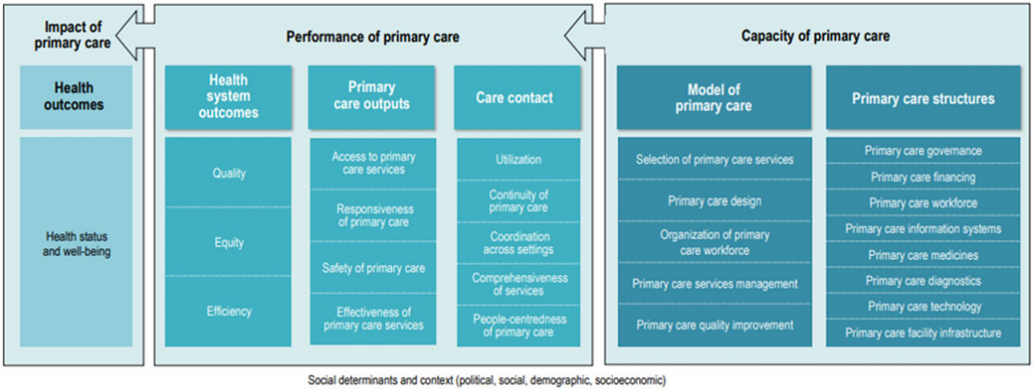
The framework underpinning PHC-IMPACT.

WHO PHC-IMPACT encompasses a comprehensive set of indicators, developed by the World Health Organization Regional Office for Europe and aiming to generate performance intelligence to strengthen and monitor the potentials of primary care for the benefit of Universal Health Coverage (Barbazza *et al.*, 2019). For the practical use by countries, the indicators are provided in a so-called Indicator Passport, structured in domains, features and indicator questions. The main components of the WHO PHC-IMPACT framework are: Capacity, Performance and Impact of primary care (from right to left in Figure 3). From broad to specific, each of these components have been structured in six domains, 26 subdomains and 63 features, which have eventually been operationalized into 139 indicators. As the purpose of SiSPC is to characterize primary care specifically at the national level, the component of ‘Capacity’ and of ‘Performance’ (as far as process indicators at system level are concerned) are most relevant here.

The evidence-base of PHC-IMPACT started from the PHAMEU systematic review which was updated by a literature review to identify frameworks and tools published between 2010 and the date of the search (2016–17) (Barbazza *et al.*, 2019).

It turned out that 21 indicators that we included from the PHAMEU list were (more or less) the same in PHC-IMPACT. Indicators that were in PHC-IMPACT, but not in the remaining PHAMEU indicator set, and satisfied our requirements, related to:

- A licensing system for family physicians (FPs) or primary care nurses
- Support for family carers
- Primary care benefit package
- Tasks of FPs formally defined
- Age balance in active FPs
- Time FP trainees spend in primary care
- Predominant practice settings of FPs
- Quality assurance mechanisms
- Patient experiences measured and published
- Quality of care processes implemented in primary care practices
- Role for representatives of citizens/patients
- Screening programmes operating via primary care
- Prescriptions by FP without specialist recommendation

These topics have been taken into consideration to be elaborated in indicators for SiSPC.

### Step 3: Searching for other frameworks and extracting relevant elements

From our scan of the literature, we evaluated nine frameworks. They revealed new domains, new dimensions and related new indicators.

Within the domain of the Structure of primary care, we added the dimension of ‘Information structures’, to explicitly cover issues like access to and use of shared medical records and a learning primary care system.

Within the (new) domain of ‘Systemic aspects of facility management’, professionalization of practice management resulted as a potential field of interest. Furthermore, ‘Composition of the primary care team’ was mentioned as potentially relevant in Systemic aspects of service delivery.

Finally, we found a few indicator fields that, in this stage, did not fit well in the proposed domains (see Table 1). We parked them – provisionally – in the domain ‘Not fitting in above Domains’. In later stages of the project, as domains further got their shape, it turned out that these indicator fields could be absorbed by one of the other domains.

**Table 1.** Overview of domains and indicator fields from other primary care-relevant frameworks

Domains	Overview of additions	Taken from:
Primary care structure	Multistakeholder participation/Engagement with private sector providers Public research funding for primary care research Tele-medicine access Access and use of shared medical records Learning Health Care System Intersectoral/health-in-all-policies Development of primary care oriented research Availability of local public health data for primary care Relations between primary care and public health Resilience of the health care system Relations between primary care and social care Policies on inequities	* OECD Health Care Quality Indicators framework (Kelley and Hurst, 2006; OECD, 2024) * WHO/Unicef Primary health care measurement framework and indicators (WHO and Unichef, 2022). * Self-assessment tool for the evaluation of essential public health operations in the WHO European Region (WHO, 2015). * Primary Health Care: a strategic framework for the prevention and control of chronic non-communicable diseases (Demaio *et al.*, 2014). * Desde el paciente: Experiencias de la Atención Primaria de Salud en América Latina y el Caribe. [From the patient: primary health care experiences in Latin America and the Caribbean] (Guanais *et al.*, 2018)
Systemic aspects of primary care facility management	(Incentives to facilitate) professionalization of practice management. Facility management capability	* WHO/Unicef Primary health care measurement framework and indicators (WHO and Unichef, 2022)
Systemic aspects of primary care delivery	Existence of care pathways for tracer conditions Multidisciplinary team-based service delivery (in particular non-physicians) Relations between primary care and public health (in particular incentives /conditions)	* WHO/Unicef Primary health care measurement framework and indicators (WHO and Unichef, 2022) * Consolidated framework for Assessing primary care organization and performance (Senn *et al.*, 2021) * WHO Self-assessment tool for the evaluation of essential public health operations in the WHO European Region (WHO, 2015) * Primary Health Care: a strategic framework for the prevention and control of chronic non-communicable diseases (Demaio *et al.*, 2014) * Marco de Monitoreo para la Salud Universal en las Américas. [Framework for monitoring universal health in America] (PAHO, 2021) * Indicadores de Coordinación asistencial entre niveles de Atención. [Indicators of clinical coordination across levels of health care] (Aller *et al.*, 2012)
Not fitting in above Domains	Life course approach to health needs: palliative/hospice care Role of civil society organizations in improving health system performance	* OECD Health Care Quality Indicators framework (OECD, 2024) * Consolidated framework for assessing primary care organization and performance (Senn *et al.*, 2021) * Primary Health Care: a strategic framework for the prevention and control of chronic non-communicable diseases (Demaio *et al.*, 2014)

The results of our third step, screening of other frameworks, are summarized in Table 1.

In screening the other frameworks, we found a category of indicators which was missing both in PHAMEU and PHC-IMPACT: indicators for the context in which primary care operates. We included ‘Context’ in our framework, be it with a special status, as these indicators do not inform about the strength of primary care in a specific country. Nevertheless, they are important in understanding why primary care is stronger in some countries than in others. Context aspects concern the broader health system; the political, social and cultural influences on the primary care system (Sidel and Sidel, 1977, Kringos *et al.*, 2013b), and the non-health care determinants of health (Whitehead, 1990, Whitehead and Dahlgren, 2006), as shown in Table 2.

**Table 2.** Overview of indicator fields on context

Indicator category	Overview of additions	Taken from:
Primary care context	Population in the country Economic features Social and cultural values, politics Welfare benefits and social protection Education (-related) resources Lifestyle data Features of the health care system overall	* OECD Health Care Quality Indicators framework (Kelley and Hurst, 2006). * Consolidated framework for assessing primary care organization and performance (Senn *et al.*, 2021). * Primary Health Care Performance Initiative framework (Veillard *et al.*, 2017).

### Step 4: Integrating topical issues

The fourth step resulted from our aim to have an up-to-date system of indicators, reflecting current (and future) challenges and needs in primary care systems that are not already incorporated in previously considered frameworks. Topical issues in primary care have been identified through a review of authoritative reports of professional and international organizations that identify insights, visions and innovations that should make primary care fit for the future.

We have identified 14 challenges and issues that play (or will play) a role in primary care. These are listed below with our decision whether each of them were covered or not by the current system of indicators. If not, one or more new indicators were added.

- *Health system resilience*, dealing with pandemics (Kruk *et al.*, 2015, Thomas *et al.*, 2020, EXPH, 2020, OECD, 2021). Resilience of primary care is covered by a number of separate indicators in the current set.
- *Environmental footprint of health care* (Lenzen *et al.*, 2020, Gonzalez-Holguera *et al.*, 2022, Klemenc Ketis and Rochfort, 2022). A new indicator was added to Primary care context.
- *Primary care in depopulating regions* (Bosmans *et al.*, 2021) and *shortage of primary care staff* (WHO, 2016a, OECD, 2020). This is covered by indicators on incentives for working in remote areas.
- *e-Health care* (OECD, 2020). Indicators related to e-health access to primary care practices and the availability of reliable online patient information were added.
- *Community involvement* (DeCamp *et al.*, 2019, Eder *et al.*, 2013, Modigh *et al.*, 2021, Sharma and Grumbach, 2017). This is covered by a number of indicators in the current set.
- *Dealing with multimorbidity* (Adan *et al.*, 2020, WHO, 2016b, Rijken *et al.*, 2018). How multimorbidity is dealt with in primary care largely varies between providers and, therefore, it can better be measured at provider or practice level. No addition is needed.
- *Mental health and primary care* (Smit *et al.*, 2020). This field is covered by an indicator on coverage in national policy documents and by an indicator on the role of primary care in access to specialized mental health care. No addition is needed.
- *Primary care and social care collaboration* (RCGP, 2019, EuroHealthNet, 2022). This field is covered by an indicator on coverage in national policy documents. No addition is needed.
- *Continuity of care out-of-hours* (RCGP, 2016, Steeman *et al.*, 2020). This is covered by an indicator on family physician involvement in out-of-hours care. No addition is needed.
- *Care team well-being, Quadruple Aim* (Bodenheimer and Sinsky, 2014). Indicators on the work-private balance were added.
- *Social prescribing* (NHS, 2019, Buck and Ewbank, 2020, WHO, 2022). We have added social prescribing under ‘Systemic aspects of service provision’.
- *Extension of family physician training duration* (RCGP, 2019, Burgmann *et al.*, 2023). This is represented by an indicator on the length of family medicine training. No addition is needed.
- *Population health approach, outreaching* (RCGP, 2019, De Maeseneer, 2017, Jiao *et al.*, 2022, NASEM, 2023, RVS, 2023). Important conditions for such approaches are registration of patients with a primary care provider (list system) and availability and exchange of relevant patient/population data. As these are covered no addition is needed.
- *Learning health system based on primary care data* (IOM, 2007, Friedman *et al.*, 2015). This is sufficiently covered as a result of the previous steps, in particular in the dimension information structures within the domain of structure of primary care. No addition is needed.

### Step 5: Processing feedback on the development process and provisional indicator system

The fifth and final step in the process of developing SiSPC consisted of consulting experts and processing their feedback. The consortium partners who implemented the PaRIS project, provided input online throughout the project and at physical meetings in September 2023 and 2024. In an early phase of the development of SiSPC, we organized a workshop at the yearly conference of EFPC, attended by some twenty participants from various countries (including e.g. Austria, Azerbaijan, Belgium, and France) and with backgrounds in general practice, policy making and research. Some of the challenges to strengthening primary care, mentioned in the workshop, were:

- Fragmentation of payment systems for primary care providers, leading to less efficient and less satisfactory organization of primary care;
- Unclear roles and responsibilities of stakeholders involved in the governance of primary care;
- Availability and implementation of self-monitoring devices for patients with chronic conditions;
- Use of primary care-generated information for surveillance;
- Organization of out-of-hours services regarding to the role of FPs and accessibility to patients.

Successes of primary care in participants’ countries were mentioned as well, in particular:

- Availability and use of guidelines;
- Availability of (shared) data from electronic medical files.

Concerning community participation in primary care, the following issues were suggested:

- Community participation is relevant both at the level of primary care practices and in health care institutions at the national level (e.g. insurance bodies);
- The importance of non-disease-specific, national patient platforms;
- The existence of legislation or national support for patient councils at primary care practice level.

In the December 2024, the draft of SiSPC was sent to experts in 34 countries. By the end of February 2025 and after only one reminder, experts from 25 countries had responded (see the acknowledgement at the end of this article). They all were positive about the initiative to develop a new system of indicators for the strength of primary care. More general remarks and suggestions that were made concern:

- **Balanced focus**

A general problem identified is whether there is enough focus on chronic disease or too much. The PaRIS project focussed on primary care for people living with chronic conditions. However, primary care also has an important role in prevention and care for acute episodes. SiSPC aims to provide a balance between attention to chronic care and other roles of primary care. Many of the aspects of strong primary care are especially relevant for people with a chronic disease (e.g., coordination and comprehensiveness of primary care). At the same time, we have included indicator items relating to prevention and acute care.

- **Focus on FPs**

The indicators that constitute SiSPC mainly refer to FPs. Ideally, all other professions active in primary care teams should also be considered. We have concluded that this is unfeasible for several reasons. First, FPs are still the backbone of primary care; secondly, equal attention to all other primary care disciplines would multiply the number of indicators, while there is little information on other professions. However, we have devoted attention to practice nurses, whose profession is increasingly important for strong primary care, especially in the care for people with a chronic disease.

- **The time dimension**

The SiSPC indicator system does not contain items on changes over time. The measurement of the SiSPC indicator system will represent the situation in the early 2020’s. A first indication of changes may come from a comparison with the PHAMEU indicator system. However, more importantly, we hope that SiSPC will be updated in line with future rounds of the PaRIS project. This will show future changes in the strength of primary care. Basically, SiSPC will provide repeated cross-sections.

- **Relevance of answering categories**

In some countries, the answering categories for indicator items do not cover the specific situation in that country. Reviewers suggested that there could be options for ‘Not applicable’, ‘Partial’ or ‘no available data’ for some indicators. Also, we should provide the option ‘Other’ with the possibility of free-text input more often. We have reviewed all indicator items to see whether additional answering options are needed. Related to this is how to deal with federal countries that have differences in their primary care system in federal states or autonomous provinces. Here, the focus remains on the communalities at country level.

- **A core set of indicators**

It was suggested to define a core set of indicators for the strength of primary care. We are not in favour of this. The indicator items refer to dimensions of strong primary care and after data collection will be combined through a statistical model. A core set of indicators may be easier for data collection but would miss out important areas. The statistical analysis will provide information on which indicator items are less relevant for an overarching dimension and that can be skipped in future data collections.

Specific additions resulting from the experts’ remarks are listed in Table 3. We left out smaller changes in wording of indicator items and in the answering options.

**Table 3.** Feedback resulting in an removal, addition or change by domain and dimension

Domain and dimension	Feedback resulting in addition or change	Reason
Context, dimension population	Added: Age dependency ratio	Additional value to percentage over 65 years
Context, dimension Social and cultural values	Added: Trust in public institutions	May be related to development of primary care
Structure of primary care, dimension governance	Removed: Indicator on contribution of independent (not for profit) civil society organizations to the primary care system	Unclear relationship with strength of primary care
Structure of primary care, dimension economic and financial conditions	Changed: Coverage of the population in resident population	‘Population’ may be unclear. Chosen for residence over citizenship
Structure of primary care, dimension economic and financial conditions	Changed: As answering options for the indicator on the basic health benefit package may differ for different categories of the population, this now only applies to the most common basic benefits package	To accommodate countries with multiple insurance modalities
Structure of primary care, dimension workforce development	Changed: guidelines on chronic conditions changed in guidelines on diseases	Whether guidelines are developed for chronic conditions or for diseases in general is not relevant
Structure of primary care, dimension information structures	Removed: Indicator on primary care publications	Difficult to measure without language bias

### The resulting full system of SiSPC indicators

As a result of the five steps, SiSPC consists of the following four domains (and dimensions within these domains):

- *Context of Primary Care* (Population, Economy, Social- and cultural values, Welfare benefits and social protection, Education (-related) resources, Life style, Health system overall);
- *Structure of Primary Care* (Governance, Economic & Financial Conditions, Workforce Development, Information Structures);
- *Systemic Aspects of Facility Management* (Scale of Primary Care Delivery, Systems /structures for Quality Assurance and Safety, Practice Management Incentives, Community Involvement);
- *Systemic Aspects of Care Delivery* (Accessibility, Comprehensiveness, Continuity, Coordination).

The full system of indicators is provided in the appendix; a selection of one item for each dimension is given in Table 4.

**Table 4.** Overview of the domains and dimensions (with the number of indicators) and one exemplary SiSPC indicator for each dimension and their response categories

Indicators of PC context (30 indicators)			
Section C.1 Population (5 indicators)		Coding/answering categories	Source(s)
Example: C.1.1	Population size	Continuous variable: # inhabitants (in mln)	Source (international databases) World Bank database https://databankfiles.worldbank.org/public/ddpext_download/POP.pdf
Section C.2 Economy (4 indicators)		Coding/answering categories	Source(s)
Example: C.2.4	Income inequality	Gini index	Source (international databases) World Bank database Gini index | Data (worldbank.org)
Section C.3 Social & cultural values (4 indicators)		Coding/answering categories	Source(s)
Example: C.3.1	Values regarding the Role of the state	Government’s vs individual responsibility: scored 1–10 (1 = completely agree with responsibility of government; 10 = completely agree with responsibility of individual). Answers: % 1–3 % 8–10	Source (international databases) World Value Survey and European Values Survey; Wave 7 (2017–2022). Q108. WVS Database (world valuessurvey.org) https://europeanvaluesstudy.eu/methodology-data-documentation/survey-2017/joint-evs-wvs/
Section C.4 Welfare benefits and social protection (3 indicators)		Coding/answering categories	Source(s)
Example: C.4.2	Protection against loss of income due to unemployment	SDG 1.3.1 Answer: % of unemployed covered against loss of income	Source (international databases) ILO ILO Data Explorer
Section C.5 Education(-related) resources (3 indicators)		Coding/answering categories	Source(s)
Example: C.5.1	Years of education	Answer: # years	Source (international databases) United Nations Development Programme.
Section C.6 Life style (3 indicators)		Coding/answering categories	Source(s)
Example: C.6.1	Smoking	Answer: % daily smokers 15+	Source (international databases) OECD Health data https://data.oecd.org/healthrisk/daily-smokers.htm (See table in folder data/Contextual indicators)
Section C.7 Health system (8 indicators)		Coding/answering categories	Source(s)
Example: C.7.5	Overall coverage of health care costs	Answer: Index SDG 3.8.1	Source (international databases) OECD Health data https://www.oecd-ilibrary.org/sites/7a7afb35-en/1/3/5/1/index.html?itemId=/content/publication/7a7afb35-en&_csp_=6cf33e24b6584414b81774026d82a571&itemIGO=oecd&itemContentType=book

Domain 1. PC structure (46 indicators)			
Dimension 1.1 Governance (12 indicators)		Coding/answering categories	Source(s)
Example: 1.1.1	A governmental health policy document issued including an explicit vision on P(H)C? *NB1:Vision means: basic principles; the role of PC in health care; priorities and future actions for PC.* *NB2: documents by stakeholders etc. are not meant here.* *NB2. The vision can be included in a broader document*	Answer: No such document (continue to 1.1.8) Yes If Yes: Year of issue (latest) …… Weblink of document(s) ……. *(Also answer 1.1.2 – 1.1.7)*	Source 1: HiT (2020–2024) - search terms: PHC – PC – primary health care – primary care – ministry of health – legislation – strategy – plan - Relevant sections: 2.4 – 2.7 – 5.3 – 6.1 – 6.2 – 7.2 – 9.1 – 9.2 Source 2: EU/OECD/Observatory Country Health Profiles Source 3: National expert /NPM (for verification/addition)
Dimension 1.2 Economic & financial conditions (8 indicators)		Coding/answering categories	Source(s)
Example: 1.2.2	Total expenditure on PC as % of total expenditure on health	Answer: … %	Source: OECD System of Health Accounts (SHA). (see document in file Data) https://www.oecd-ilibrary.org/sites/7a7afb35-en/1/3/4/1/index.html?itemId=/content/publication/7a7afb35-en&_csp_=6cf33e24b6584414b81774026d82a571&itemIGO=oecd&itemContentType=book
Dimension 1.3 Workforce development (22 indicators)		Coding/answering categories	Source(s)
Example: 1.3.2	Are FPs obliged to participate in continuous professional development (CPD)? (e.g. in a system of gaining points)	Yes No	Source 1: HSC Survey (Q.43) Source 2: EURACT CME/CPD database (account needed) (39 European countries) https://www.euract.eu/country-database-entries/index/93c7b61e-25f3-4dde-89b6-2ce13168484a Source 3: National expert/NPM
Dimension 1.4 Information structures (4 indicators)		Coding/answering categories	Source(s)
Example: 1.4. 1	Are clinical patient records from FP/PC used at regional or local level to identify health needs or priorities for health policy?	Routinely (e.g. in health statistics) Incidentally Seldom or never	Source: National expert/NPM

Domain 2. Systemic aspects of facility management (7 indicators)			
Dimension 2.1 Scale of PC delivery (1 indicator)		Coding/answering categories	Source(s)
Example: 2.1.1	Which percentage of FPs are working in the following practice settings? *(NB: other PC disciplines: e.g. physiotherapist; social worker; speech therapist)*	1 FP (with or without a nurse): …… % 2 or more FPs (with or without a nurse): …… % 1 or 2 FPs (with or without a nurse) plus other PC discipline(s): …… % 3 or more FPs (with or without a nurse) plus PC discipline(s) …… % *o* FPs in other settings, namely ……. …. %	Source: National expert/NPM
Dimension 2.2 Systems/ structures for quality assurance and safety (4 indicators)		Coding/answering categories	Source(s)
Example: 2.2.4	Are patient experiences measured at facility level?	No (or very rarely) Yes, incidentally *o* Yes, widespread	Source: National expert/NPM
Dimension 2.3 Practice management incentives (1 indicator)		Coding/answering categories	Source(s)
Example: 2.3.1	Is an allocated budget available for PC/FP practices to pay a (part-time) practice manager?	Yes No	Source: National expert/NPM
Dimension 2.4 Community involvement (1 indicator)		Coding/answering categories	Source(s)
Example: 2.4.1	To what extent do citizens/ patient representatives have any formal role in the areas specified?	Training/education for patients Yes No Membership in PC advisory boards at community level (e.g. council boards) Yes No Membership in supervisory boards of PC facilities Yes No	Source: National expert/NPM
Domain 3. Systemic aspects of care delivery (17 indicators)			
Dimension 3.1 Accessibility (6 indicators)		Coding/answering categories	Source(s)
Example: 3.1.1	The total number of (directly accessible) active FPs available per 100 000 population	FPs: …. per 100.000	Source 1: (partly/t.b.s) WHO/HlthRes-DB. European database on human and technical resources for health - European Health Information Gateway (who.int) Source 2: National expert/NPM
Dimension 3.2 Comprehensiveness (6 indicators)		Coding/answering categories	Source(s)
Example: 3.2.6	To what extent is social prescribing in PC practices recognized? *(Social prescribing means that FPs can refer patients to non-clinical social programmes in the community. The focus can be on i.a. income, health food, sports, housing, social activation, informal care support)*	Social prescribing is formally recognized and increasingly practiced Social prescribing is being discussed but not (or hardly) practiced Social prescribing is currently (practically) unknown	Source 1: Literature; answers for 12 countries Source 2: National expert/NPM (for other countries)
Dimension 3.3 Continuity (3 indicators)		Coding/answering categories	Source(s)
Example: 3.3.3	Are people registered with a FP practice?	Yes almost) the whole population (> 95%) Y/N the majority (> 50%) Y/N less than 50% Y/N No	Source 1: OECD/HSC Survey (q.37) Source 2: HiT (Ch5) Source 3: National expert/NPM
Dimension 3.4 Coordination (2 indicators)		Coding/answering categories	Source(s)
Example: 3.4.1	To what extent do PC physicians control access to (medical) specialist care?	PC physician referral is compulsory to access most types of (medical) specialist care (except in case of emergency) Y/N PC physician referral is compulsory to access restricted types of (medical) specialist care (except in case of emergency) Y/N Patients have financial incentives to obtain a PC physicians’ referral (e.g. reduced co-payments), but direct access is always possible Y/N There is no need and no incentive to obtain PC physician referral Y/N	Source 1: OECD/HSC Survey (q.38) Source 2: HiT Source 3: National expert/NPM

## Discussion

**Figure 4.**
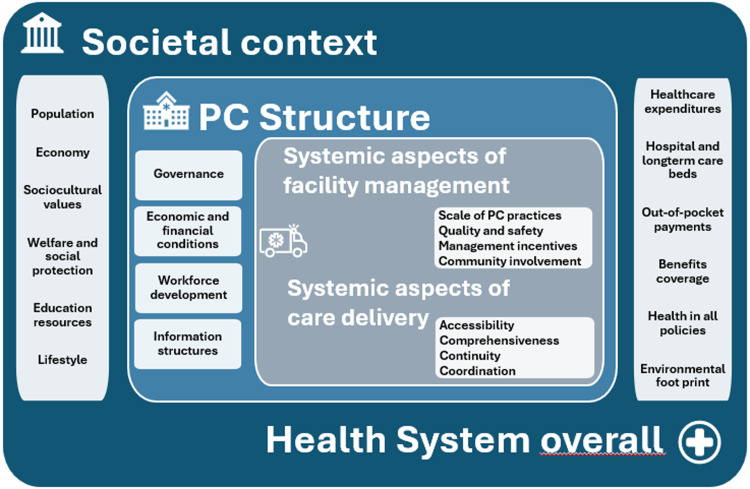
The SiSPC framework.

We have developed the new indicator system SiSPC in a number of steps. SiSPC is based on existing frameworks. The (future) measurement of the items will provide continuity with the past and make comparison over time possible. Topical issues and challenges were added to provide an update with new indicators for the strength of primary care. The resulting indicator system is summarized in Figure 4.

The system of indicators provided by SiSPC will be an important tool for researchers to measure the strength of primary care. The results of the measurement of the SiSPC indicators can be used by policy-makers to monitor the primary care system in their country. As latest developments were taken into account, it measures ‘strength’ in line with current expectations of primary care. SiSPC can be used to satisfy various needs for information, such as:

- Describing, at country level, the state of affairs of primary care (particularly in high- and middle-income countries) and to monitor its development in the future (with repeated measurement);
- Analyzing whether and how the strength of primary care is related to the country context;
- Analyzing whether and how the strength of primary care is associated with outcomes of primary care, as measured, e.g., at provider level and at patient level.
- Showing changes in the strength of primary care between 2005 and 2023 (by comparing SiSPC with PHAMEU).

By its exclusive focus on the structural or systemic features of primary care systems, SiSPC contributes to clarity of information relevant to different levels of health care. In other tools and frameworks, indicators relating to the system level, the provider level and the patient level are often mixed. This hinders a clear analytical separation of issues that are at play at these different levels. Such a separation, however, is vital for policymakers, as policy levers employed to improve the primary care system often operate level-specific. A clear empirical analysis of the different areas where improvements can be made, is possible by combining SiSPC data with, for instance, data from surveys among providers and patients in a multi-level design (see for example Figure 1 in (Groenewegen *et al.*, 2024)).

The practical use of SiSPC in research has been facilitated by a focus on the feasibility of data collection. Many data can be collected centrally from international sources (e.g. from databases of the OECD, World Health Organization, European Union, World Bank and from international publications, such as the Health Systems in Transition series from the European Observatory on Health Systems and Policies), as witnessed by the entries in the column ‘sources’ in the overview of SiSPC. This will reduce the burden of data collection at country level considerably. Furthermore, experts in the countries have been asked to provide their feedback on the draft final system of indicators, particularly on the clarity of the indicator questions and the feasibility of data collection.

In developing SiSPC, we seized the opportunity to realize it alongside the PaRIS project. OECD aims to develop PaRIS into a regular data collection in an increasing number of countries. Updates and data collection for SISPC could follow the future rounds of PaRIS. However, even though the indicator system is ready-for-use, a large-scale data collection and analyzing the data (e.g.) every five years may be a challenge.

Further to previously mentioned strong points of SiSPC we point to its basis in existing validated frameworks, in particular PHAMEU and PHC-IMPACT. At the same time, we have not performed a new literature review to assess the evidence-base for newly developed indicators. As a second-best option we relied on previous work by the authors of the main frameworks that we used in the development of SiSPC, and on the Position Paper of EFPC on the organization of primary care (Akman *et al.*, 2022).

The focus in SiSPC is on family physicians. We are aware that primary care is broader and also includes the work of nurses and professionals within primary care. Family physicians are (still) the core providers of primary care. However, we have given attention to their relationships with other primary care providers and social care. Another limitation is that we focused largely on Western countries and OECD member states, which are mostly higher-middle and high-income countries. This was done first of all for reasons of comparability. Despite the heterogeneity that generally characterizes health care systems, those in these countries are relatively well comparable as they share a basic institutional set-up. Another reason was that SiSPC has been developed in the context of the PaRIS project and on the basis of PHAMEU. The first implementation, in terms of data collection and analytical use of the data, will be in the countries participating in PaRIS and those in PHAMEU. Nevertheless, it will be important and interesting to assess the usability and validity of the SiSPC in lower- and middle-income countries, in line with developments in their primary care systems. SiSPC’s focus is on the system level and on national level. However, there may be subnational, regional variation in primary care systems in some countries. These are difficult to handle in international comparative research and can be better studied within countries.

Our next step is the collection of data to measure the indicator items. After this is done, it is possible to analyze whether and how the strength of primary care is associated with the assumed outcomes of strong primary care. The data collection has started and we hope to report about it soon.

## Supporting information

Boerma et al. supplementary material Boerma et al. supplementary material

## Data Availability

No new data was generated or analyzed in support of this research.

## Supplementary material

The supplementary material for this article can be found at https://doi.org/10.1017/S1463423625100509
