# Supplementary material for: Measuring the strength of primary care: development of a new system of Structural Indicators for the Strength of Primary Care – SiSPC
Source: Prim Health Care Res Dev. 2025 Sep 30;26:e85. doi: 10.1017/S1463423625100509 (PMC12555084; doi:10.1017/S1463423625100509)
Supplement: Boerma et al. supplementary material [file S1463423625100509sup001.docx]

Appendix: Overview of all SiSPC indicators, answering categories and sources of information

Overview of indicators, answering categories and sources of information

Abbreviations:

FP = Family physician

PC = Primary care

HSPM = Health Systems Performance Monitor

HiT = Health Systems in Transition Series (European Observatory on Health Systems and Policies)

HSC= OECD Health System Characteristics survey

| **Indicators of PC Context** (not on strength of primary care)(30 indicators) | | | |
| --- | --- | --- | --- |
| Section C.1 Population  (5 indicators) | | Coding / answering categories | Source(s) |
| C.1.1 | Population size | Continuous variable:   - # inhabitants (in mln) | Source (internat. databases)  WorldBank database  [https://databankfiles.worldbank.org/public/ddpext_download/ POP.pdf](https://databankfiles.worldbank.org/public/ddpext_download/%20POP.pdf) |
| C.1.2 | Population density | Continuous variable   - # inhabitants per km^2^ | Source (internat. databases)  Worldbank database [Population density (people per sq. km of land area) \| Data (worldbank.org)](https://data.worldbank.org/indicator/EN.POP.DNST) |
| C.1.3 | Age distribution | - % of population 65 /+ | Source (consortium)  Worldbank database [Population ages 65 and above (% of total population) \| Data (worldbank.org)](https://data.worldbank.org/indicator/SP.POP.65UP.TO.ZS?name_desc=false&view=chart) |
| C1.4 | Age dependency ratio | ….. % of dependents per 100 working-age population | Source: <https://databank.worldbank.org/metadataglossary/gender-statistics/series/SP.POP.DPND#:~:text=Age%20dependency%20ratio%20is%20the,per%20100%20working%2Dage%20population> |
| C.1.5 | Urbanicity | - % of population living in urban areas (as defined by national statistical offices) | Source (internat. databases)  WorldBank database  <https://data.worldbank.org/indicator/SP.URB.TOTL.IN.ZS> |
| Section C.2 Economy  (4 indicators) | | Coding / answering categories | Source(s) |
| C.2.1 | Structure of the economy; added value per sector | Answer:   - added value to GDP:   - % primary sector   - % secondary sector   - % tertiary sector | Source (internat. databases)  Worldbank database  Primary: [Agriculture, forestry, and fishing, value added (% of GDP) \| Data (worldbank.org)](https://data.worldbank.org/indicator/NV.AGR.TOTL.ZS?view=chart)  Secondary: [Industry (including construction), value added (% of GDP) \| Data (worldbank.org)](https://data.worldbank.org/indicator/NV.IND.TOTL.ZS?view=chart)  Tertiary: [Services, value added (% of GDP) \| Data (worldbank.org)](https://data.worldbank.org/indicator/NV.SRV.TOTL.ZS?view=chart) |
| C.2.2 | Unemployment | - Average % last 5 years | Source (internat. databases)  Worldbank database  [World Development Indicators \| DataBank (worldbank.org)](https://databank.worldbank.org/reports.aspx?source=2&series=SL.UEM.TOTL.NE.ZS&country=) |
| C.2.3 | Gross national income (GNI) per capita | - In PPP $ | Source (internat. databases)  United Nations Development Programme (UNDP). |
| C.2.4 | Income inequality | - Gini index | Source (internat. databases)  WorldBank database [Gini index \| Data (worldbank.org)](https://data.worldbank.org/indicator/SI.POV.GINI) |
| Section C.3 Social & cultural values (4 indicators) | | Coding / answering categories | Source(s) |
| C.3.1 | Values regarding the Role of the state | Government’s vs individual responsibility: scored 1-10  (1 = completely agree with responsibility of government;  10 = completely agree with responsibility of individual).  Answers:  - % 1-3  - % 8-10 | Source (internat. databases)  World Value Survey and European Values Survey; Wave 7 (2017-2022). Q108.  [WVS Database (world valuessurvey.org)](https://www.worldvaluessurvey.org/wvs.jsp) https://europeanvaluesstudy.eu/methodology-data-documentation/survey-2017/joint-evs-wvs/ |
| C.3.2 | Family values | Child’s duty to take care of ill parent (degrees of agreement).  Answer:   - - % (strongly) agree | Source (internat. databases)  World Value Survey and European Values Survey; Wave 7 (2017-2022). Q38.  [WVS Database (world valuessurvey.org)](https://www.worldvaluessurvey.org/wvs.jsp) https://europeanvaluesstudy.eu/methodology-data-documentation/survey-2017/joint-evs-wvs/ |
| C.3.3 | Government participation of left-wing parties | Number of years in government in the period 2007-2021 (last 15 years)  Answers (to be calculated):   - 1.00=100% of years left wing parties; - 0,75= >66,6%; - 0,50= 66,6-33,3%; - 0,25= <33,3%.   Variable: GOV_left1. | Source (internat. databases)  Inst of Pol Sc, Univ of Bern (Comparative political dataset) [Data – Comparative Political Data Set (cpds-data.org)](https://cpds-data.org/data/) |
| C.3.4 | Trust in public institutions | ….. % of population who indicate trust in their national government (0-10 scale) | Source  <https://www.oecd.org/en/topics/trust-in-government.html> |
| Section C.4 Welfare benefits and social protection  (3 indicators) | | Coding / answering categories | Source(s) |
| C.4.1 | Social expenditure | Public social spending  Answer:   - As % of GDP | Source (internat. databases)  OECD database [Social Expenditure - Aggregated data (oecd.org)](https://stats.oecd.org/Index.aspx?DataSetCode=SOCX_AGG) |
| C.4.2 | Protection against loss of income due to unemployment | SDG 1.3.1  Answer:   - % of unemployed covered against loss of income | Source (internat. databases)  ILO [ILO Data Explorer](https://www.ilo.org/shinyapps/bulkexplorer3/?lang=en&id=SDG_0131_SEX_SOC_RT_A) |
| C.4.3 | Effectiveness of pension schemes: net pension replacement rate.  *(Defined as the individual net pension entitlement divided by net pre-retirement earnings; taking into account personal income taxes and social security contributions paid by workers and pensioners).* | Answer:   - Pension as % of pre-retirement earnings (by gender). | Source (internat. databases)  OECD (2024), Net pension replacement rates. doi: 10.1787/4b03f028-en (Accessed on 01 February 2024).  [Pensions - Net pension replacement rates - OECD Data](https://data.oecd.org/pension/net-pension-replacement-rates.htm#:~:text=The%20net%20pension%20replacement%20rate,paid%20by%20workers%20and%20pensioners.) |
| Section C.5 Education(-related) resources  (3 indicators) | | Coding / answering categories | Source(s) |
| C.5.1 | Years of education | Answer:   - # years | Source (internat. databases)  United Nations Development Programme (UNDP). |
| C.5.2 | Human Development Index (HDI) | Answer:   - index | Source (internat. databases)  United Nations Development Programme (UNDP). |
| C.5.3 | Internet access | Answer:   - % of households with internet access | Source (internat. databases)  Eurostat EU survey on the use of Information and Communication Technologies (ICT) in households and by individuals [https://ec.europa.eu/eurostat/databrowser/view/isoc_ci_in_h/ default/table?lang=en](https://ec.europa.eu/eurostat/databrowser/view/isoc_ci_in_h/%20default/table?lang=en) |
| Section C.6 Life style  (3 indicators) | | Coding / answering categories | Source(s) |
| C.6.1 | Smoking | Answer:   - % daily smokers 15+ | Source (internat. databases)  OECD Health data  <https://data.oecd.org/healthrisk/daily-smokers.htm>  (See table in folder data/Contextual indicators) |
| C.6.2 | Alcohol use | Answer:   - # litres annual sales of pure alcohol per person aged 15+ | Source (internat. databases)  OECD Health data  <https://data.oecd.org/healthrisk/alcohol-consumption.htm>  (See table in folder data/Contextual indicators) |
| C.6.3 | Population overweight or obese | Answer:   - % population overweight or obese aged 15+ (self-reported or measured) | Source (internat. databases)  OECD Health data  <https://data.oecd.org/healthrisk/overweight-or-obese-population.htm> |
| Section C.7 Health system  (8 indicators) | | Coding / answering categories | Source(s) |
| C.7.1 | Health expenditure | Answer:   - % of GDP | Source (internat. databases)  WHO Global Health Expenditure Database (GHED) <https://apps.who.int/nha/database/Select/Indicators/en> |
| C.7.2 | Hospital beds | Answer:   - #/1000 population | Source (internat. databases)  <https://data.oecd.org/healtheqt/hospital-beds.htm> |
| C.7.3 | Long-term care beds | Answer:   - #/1000 population | Source (internat. databases)  OECD health statistics  <https://stats.oecd.org/Index.aspx?QueryId=30142> |
| C.7.4 | Non-public sources of revenue (Out of pocket payments OoP; Voluntary health insurance VHI) | Answers:   - OoP as % of current health expenditure - VHI as % of current health expenditure | Source (internat. databases)  WHO Global Health Expenditure Database (GHED) <https://apps.who.int/nha/database/Select/Indicators/en> |
| C.7.5 | Overall coverage of health care costs | Answer:   - Index SDG 3.8.1 | Source (internat. databases)  OECD Health data https://www.oecd-ilibrary.org/sites/7a7afb35-en/1/3/5/1/index.html?itemId=/content/publication/7a7afb35-en&_csp_=6cf33e24b6584414b81774026d82a571&itemIGO=oecd&itemContentType=book |
| C.7.6 | Situation of ‘Health-in-all-policies’ (HiAP) in the country | Answer (most appropriate):   - (1) Very little or no HiAP awareness, action or whatsoever - (2) Emerging (there is a governmental vision; interest and intersectoral contacts but no formal commitment to develop HiAP). - (3) Progressing (there is formal commitment to proceed with HiAP; committees or task forces exist; but governance structures and implementation plans are in an early stage) - (4) Established: governance and implementation mechanisms work well; HiAP is embedded as a recognised way of working; e.g. health impact analyses; health lens analyses) | Source 1: (internat. databases):  HiTs, text from section 2.5  (See subfolder Contextual indicators in folder Data)  Source 2: (optional) National expert/NPM |
| C.7.7 | To what extent is environmental footprint of health care a reality?  (one answer) | Answer (most appropriate):   - (1) No awareness, action, estimates or whatsoever on environmental footprint of health care - (2) Awareness but no or little action in this respect - (3) A vision on environmental footprint has been laid down in a policy document by government or professional organisation - (4) Estimates / calculations of the environmental footprint of health care facilities are available | Source 1 (internat. databases)  See: “Indicators C7.7 / C7.8 Environmental Footprint, Data Sources per country”; in folder Data  Source 2:  National expert /NPM / nat. source |
| C.7.8 | Are estimates or calculations of the environmental footprint of PC facilities specifically available? | Answer:   - Yes - No | Source 1 (internat. databases)  See: “Indicators C7.7 / C7.8 Environmental Footprint, Data Sources per country”; in folder Data  Source 2:  National expert/NPM |
| **DOMAIN 1. PC Structure** (46 indicators) | | | |
| DIMENSION 1.1 Governance (12 indicators) | | Coding / answering categories | Source(s) |
| 1.1.1 | A governmental health policy document issued including an explicit vision on P(H)C?    *NB1:Vision means: basic principles; the role of PC in health care; priorities and future actions for PC.*  *NB2: documents by stakeholders etc. are not meant here.*  *NB2. The vision can be included in a broader document* | Answer:   - No such document *(continue to 1.1.8)* - Yes   If Yes:   - Year of issue (latest) …… - Weblink of document(s) ……..   *(Also answer 1.1.2 – 1.1.7)* | Source 1: HiT (2020 – 2024)   - search terms: PHC – PC – primary health care – primary care – ministry of health – legislation – strategy – plan - Relevant sections: 2.4 – 2.7 – 5.3 – 6.1 – 6.2 – 7.2 – 9.1 – 9.2   Source 2: EU/OECD/Observatory Country Health Profiles  Source 3: National expert /NPM (for verification / addition) |
| 1.1.2 | *Re. 1.1.1 (if ‘yes’)*  What is the status of the document in 1.1.1? | Answer: (which applies)   - (1) Policy paper - (2) Law/ regulation/ directive - (3) Other: namely ….. | Source 1: HiT (2020 – 2024)  Source 2: National expert /NPM (details in 1.1.2 – 1.1.7 may not be available in HiTs) |
| 1.1.3 | *Re. 1.1.1 (if ‘yes’)*  Has a policy on cooperation among services and providers within PC explicitly been mentioned? | Answer:   - Yes - No | Source 1: HiT (2020 – 2024)  Source 2: National expert /NPM  The document used in 1.1.1 to be searched with (combinations of) these terms:  - collaboration  - teamwork  - network  - interdisciplinary  - integration |
| 1.1.4 | *Re. 1.1.1 (if ‘yes’)*  Has a policy on cooperation between PC and specialised mental health services explicitly been mentioned? | Answer:   - Yes - No | Source 1: HiT (2020 – 2024)  Source 2: National expert /NPM  The document in 1.1.1 to be searched with (combinations of) these terms:  - teamwork  - mental healthcare  - collaboration |
| 1.1.5 | *Re. 1.1.1 (if ‘yes’)*  Has a policy on cooperation between PC and social services explicitly been mentioned? | Answer:   - Yes - No | Source 1: HiT (2020 – 2024)  Source 2: National expert /NPM  The document in 1.1.1 to be searched with (combinations of) these terms:  - collaboration  - social services  - community services |
| 1.1.6 | *Re. 1.1.1 (if ‘yes’)* Has a policy on cooperation between PC and Public Health explicitly been mentioned? | Answer:   - Yes - No | Source 1: HiT (2020 – 2024)  Source 2: National expert /NPM  The document in 1.1.1 to be searched with (combinations of) these terms:  - public health  - public health services  - collaboration |
| 1.1.7 | *Re. 1.1.1 (if ‘yes’)*  Has a policy to avoid inequities been mentioned (i.a. concerning particular groups in the population) | Answer:   - Yes - No | Source 1: HiT (2020 – 2024)  Source 2: National expert /NPM  The document in 1.1.1 to be searched with (combinations of) these terms:  - inequity  - discrimination  - migrants  - minority group  - health literacy  - women |
| 1.1.8 | Have (major) responsibilities for PC been decentralized to regional or local level? | Answer:   - Yes - No | Source 1: HiT (section 2.3)  Source 2: HSPM / Country Health Profile [HSPM (who.int)](https://eurohealthobservatory.who.int/monitors/health-systems-monitor/countries-hspm)  Source 3: (optional): National expert / NPM |
| 1.1.9 | If state inspection on health care exists, does it have a specific unit for PC? | Answers:   - N.a. (no state inspection) - Yes - No | Source: National expert / NPM |
| 1.1.10 | What is the role of patient organisations in aspects A-E of health care policy making? | Answers A-E:   - (A) In making any key decision in health policy   - No   - As an observer   - Consulted   - As voting member - (B) In any expert panel or workshop at the Ministry of Health:   - No   - As an observer   - Consulted   - As voting member - (C) In any Health technology assessment (HTA) procedure for new treatment options   - No   - As an observer   - Consulted   - As voting member - (D) In any health decision making in the national parliament   - No   - As an observer   - Consulted   - As voting member - (E) In any ethics committees for clinical trials   - No   - As an observer   - Consulted   - As voting member | Source 1: HiT section 7.1.2  Source 2: National expert / NPM  For France and Italy:  Souliotis K, Agapidaki E, et al. Assessing Patient Organization Participation in Health Policy: A Comparative Study in France and Italy. Int J Health Policy Manag. 2018 Jan 1;7(1):48-58. doi: 10.15171/ijhpm.2017.44. PMID: 29325402; PMCID: PMC5745867.  Answering categories get value 0, 1, 2 or 3.  (Indicator score is sum of answers). |
| 1.1.11 | Have any laws/regulation pertaining to informed consent been implemented (also applicable to treatment in PC)? | Answer:   - Yes - No | Source 1: HiT, section 2.8.3 |
| 1.1.12 | Have any laws/regulation pertaining to a procedure to process patient complaints been implemented (also applicable to PC facilities)? | Answer:   - Yes - No | Source 1: HiT, section 2.8.3  Source 2: National expert / NPM |
| DIMENSION 1.2 Economic & Financial Conditions  (8 indicators) | | Coding / answering categories | Source(s) |
| 1.2.1 | At the national level, does PC have a budget that can be distinguished from other levels of care (e.g. specialist care)? | Answer:   - Yes - No | Source: National expert / NPM |
| 1.2.2 | Total expenditure on PC as % of total expenditure on health | Answer:   - … % | Source: OECD System of Health Accounts (SHA). (see document in file Data) https://www.oecd-ilibrary.org/sites/7a7afb35-en/1/3/4/1/index.html?itemId=/content/publication/7a7afb35-en&_csp_=6cf33e24b6584414b81774026d82a571&itemIGO=oecd&itemContentType=book |
| 1.2.3 | Total expenditure on prevention and public health as % of total expenditure on health | Answer:   - … % | Source: WHO Global Health Expenditure Database (GHED)  <https://apps.who.int/nha/database/Select/Indicators/en> |
| 1.2.4 | What is the most frequent payment system for FPs/PC providers?  (indicate the most frequent payment mode in case of a single mode of payment or the most frequent combination of single payment modes) | Answer:   - Salary - Capitation - Fee-for-service - Pay-for-performance - Bundled payments - Global budget - Other, please specify | Source 1: OECD HSC Survey (Q.18b)  (see document in file Data)  Source 2: HiT (section 3.7 Payment mechanisms)  Source 3: National expert / NPM |
| 1.2.5 | Is any of the following support available for carers/family carers? | - In cash (e.g. care allowance, paid care leave, attendance allowance)   - Yes / No - In kind (e.g. vouchers, respite services, social insurance contributions, unpaid care leave, day/night care services, community care services in general)   - Yes / No | Source 1:European Social Policy Network ESPN  LTC report; country profiles (see document in file Data)  <https://ec.europa.eu/social/BlobServlet?docId=24080&langId=en>  Search term: ‘’ informal care’  Source 2:HiT (section 5.9 Services for informal carers)  Source 3: National expert / NPM |
| 1.2.6 | What % of the resident population obtains basic PC coverage through the following modes? | - Automatic PC coverage (e.g. based on residence)   - ….. % - Compulsory/mandatory PC coverage, based on payment of a specific contribution or premium (by individuals or households)   - ...% | Source 1: HSC Survey (mind: specific PC coverage)(Q.1)  (see document in file Data)  Source 2: HiT (sections 3.3.1 Coverage)  Source 3: National expert / NPM |
| 1.2.7 | To what extent are the following FP services included in the (most common) basic health benefits package? | FP office consultations and home visits   - Free at point of care   - Yes / No - Subject to a co-payment per service   - Yes / No - Subject to a co-payment as % of the price   - Yes / No - Not part of the basic benefit package   - Yes / No   FP prescribed medicines   - Free at point of care   - Yes / No - Subject to a co-payment per service   - Yes / No - Subject to a co-payment as % of the price   - Yes / No - Not part of the basic benefit package   - Yes / No | Source 1  HSC Survey, Q.12 re.   - Outpatient care contacts /Primary physician - Medicines (not PC only)   Source 2  WHO Europe (Can people afford to pay for health care)  Source 3  National expert / NPM |
| 1.2.8 | Are FPs/PC practices remunerated for online consultations? | - Yes - No | Source: National expert / NPM |
| DIMENSION 1.3 Workforce Development (22 indicators) | | Coding / answering categories | Source(s) |
| 1.3.1 | What is the (estimated) share of general physicians who did *not complete a family medicine specialisation* in the provision of first contact care?  *NB: Residents in Family Medicine are not meant here.* | - Important (>20% estimated) - Marginal (5-20%) - Insignificant / absent (<5%) | Source: National expert / NPM |
| 1.3.2 | Are FPs obliged to participate in continuous professional development (CPD)? (e.g. in a system of gaining points) | - Yes - No | Source 1: HSC Survey (Q.43)  Source 2: EURACT CME/CPD database (account needed) (39 European countries) <https://www.euract.eu/country-database-entries/index/93c7b61e-25f3-4dde-89b6-2ce13168484a>  Source 3: National expert / NPM |
| 1.3.3 | Do national association(s) or college(s) of FPs exist which have a focus on professional development, medical education and/or scientific activities?  *(NB: focus beyond defending material interest).* | - Yes - No   If yes: weblink | Source 1: WONCA Europe; member organizations (122 in 102 countries) [Member Organisations \| WONCA Europe](https://www.woncaeurope.org/page/member-organisations) (with links to each member)  Source 2: National expert / NPM |
| 1.3.4 | Do national organisation(s) (or/and nurses scientific bodies) of PC nurses exist which have a focus on professional development and education and/or scientific activities?  *(NB: focus beyond defending material interest; this focus may be represented in a broader professional organisation).* | - Yes - No   If yes: weblink | Source 1: [International Council of Nurses (ICN) \| ICN - International Council of Nurses](https://www.icn.ch/)  Source 2 National expert / NPM |
| 1.3.5 | Are evidence-based national clinical practice guidelines/clinical protocols/standards available for the management (diagnosis and treatment) of diseases in PC? | - Yes - No   If yes: how have these been developed?   - By the PC profession (e.g. professional FP association or college) Yes/No - Otherwise (e.g. by medical specialists; Ministry of Health) Yes/No | Source: National expert / NPM |
| 1.3.6 | Which of the following health professionals are *directly accessible, without a referral* in PC? | - Family physician Y/N - Midwife Y/N - PC nurse Y/N - District (community) nurse Y/N - Social worker Y/N - Psychologist Y/N - Mental health nurse Y/N - Physiotherapist Y/N - Dietician / nutritionist Y/N - Occupational therapist Y/N - Speech therapist Y/N | Source: National expert / NPM |
| 1.3.7 | Have tasks/duties of FPs been formally defined, by the government or professional bodies? | - Yes - No | Source: National expert / NPM |
| 1.3.8 | Do mechanisms exist to encourage FPs to work in underserved, remote and/or rural areas? | - Yes - No   If yes, what kind of mechanisms:   - Compulsory service requirements in rural and remote areas Yes/No - Scholarships, bursaries or other education subsidies Yes/No - Financial incentives (e.g. hardship allowances, grants for housing, transportation) Yes/No - Other, namely :……. Yes/No | Source 1: HiT (sections 4.2.2 Trends in health workforce; 5.3 Primary care)  Source 2: National expert / NPM |
| 1.3.9 | Which of the following policies exist to address identified shortages of FPs?  (more answers possible) | - Not applicable (no shortages)   - Yes / No - No particular policy   - Yes / No - Increase training capacity   - Yes / No - Prolong working time for physicians   - Yes / No - Targeted immigration policies   - Yes / No - Incentives to foster the take-up of general practice   - Yes / No - Introduction or expansion of non-physician practitioner roles   - Yes / No - Financial incentives to correct geographic maldistribution   - Yes / No - Other, namely_________   - Yes / No | Source 1: HSC Survey (Q.44)  Source 2: National expert / NPM |
| 1.3.10 | Are data available on FP workforce capacity needs and development in the future? | - Yes - No   If yes: source / weblink | Source: National expert / NPM |
| 1.3.11 | How does the gross annual income of a mid-career FP relate to the gross annual income of the following mid-career medical specialists of the same age? | Compared to FPs:   - Cardiologist income is: much lower / lower / equal / higher / much higher - Obstetrician/gynaecologist income is: much lower / lower / equal / higher / much higher - General internist income is: much lower / lower / equal / higher / much higher | Source 1: OECD StatHealth (data for 13 countries)  Source 2: National expert / NPM |
| 1.3.12 | What is the age balance between the number of younger and older practicing FPs (under 35 and over 55)? | - Under 35 …… % - Over 55 ……. % | Source 1: Eurostat  Source 2: National expert / NPM |
| 1.3.13 | Which % of medical universities (or universities with a medical faculty) offer a postgraduate programme in General practice/Family Medicine? | - ……. % - Not applicable; postgraduate programme not is organised by (medical) universities, namely ……….. ) | Source: National expert / NPM |
| 1.3.14 | How much time do family medicine trainees spend practicing in a PC/FP practice during postgraduate specialisation? | - ……. months | Source 1: EURACT CME/CPD database (39 European countries) <https://www.euract.eu/country-database-entries/index/93c7b61e-25f3-4dde-89b6-2ce13168484a>  Source 2: National expert / NPM |
| 1.3.15 | What % of all medical graduates have graduated as a FP? | - ……. % of all medical graduates (latest available year) | Source 1: EURACT Specialist training database (39 European countries) <https://www.euract.eu/country-database-entries/index/93c7b61e-25f3-4dde-89b6-2ce13168484a>  Source 2: National expert / NPM |
| 1.3.16 | Is there professional training specifically for the following two types of PC nurses? | - District/community nurses: Y/N - PC/FP practice nurses: Y/N | Source: National expert / NPM |
| 1.3.17 | Do nurses work in advanced roles (e.g as nurse practitioner, nurse specialist or diabetes nurse) in PC in the treatment of people living with chronic conditions?  *(NB: advanced tasks are beyond the traditional scope of practice, including i.a. diagnosis, treatment, prescribing, first point of contact, responsibility for a group of patients).* | - Yes, nurses are working in advanced roles in PC - Yes on a limited scale (e.g. in some regions/practices, in pilots or incidental projects) - No nurses are working in advanced roles | Source 1: OECD/HSC Survey (Q.47) |
| 1.3.18 | Is a journal on family medicine/general practice being published in this country? | - Yes - No   If yes: weblink | Source 1: SJR ranking (see document in file Data)  Source 2: NLM search of FM/PC journals (see document in file Data)  Source 3: Google search (‘journal of family medicine and primary care in [country]’)  Source 4: National expert / NPM |
| 1.3.19 | Is a professional journal on PC nursing being published in this country? | - Yes - No   If yes: weblink | National expert / NPM |
| 1.3.20 | Which % of active FPs is currently working part-time (≤ 4 days per week, excl. out of hours duties)? | - …… % - Not known | National expert / NPM |
| 1.3.21 | Does the postgraduate curriculum in family medicine / general practice offer the possibility for part-time residents? | - Yes - No | National expert / NPM |
| 1.3.22 | What is the duration of the postgraduate training in family medicine / general practice? | - ..… years | Source 1: internet search  Source 2: National expert / NPM |
| DIMENSION 1.4 Information Structures (4 indicators) | | Coding / answering categories | Source(s) |
| 1.4. 1 | Are clinical patient records from FP/PC used at regional or local level to identify health needs or priorities for health policy? | Routinely (e.g. in health statistics)  Incidentally  Seldom or never | Source: National expert / NPM |
| 1.4.2 | Share of FPs who indicated the following state of telehealth use in their practice: | 0 = not aware  1 = do not have it  2 = have it and do not use it  3 = use it occasionally  4 = use it routinely  Scale score ….. | Source 1: EC (2018), Benchmarking Deployment of eHealth among General  Practitioners ­ 2018, European Union.  Source 2: Outside EU: National expert /NPM  Switzerland: Data from the CWF IHP survey, published in Obsan report «Ärztinnen und Ärzte in der Grundversorgung – Situation in der Schweiz und im internationalen Vergleich». (response options differ). |
| 1.4.3 | Are primary care data used regularly to report on health care quality or health system performance? | Yes  No | Source 1: OECD Survey of Health Data Use and Governance, 2020.  Source 2: (Non-OECD members) National expert / NPM) |
| 1.4.4 | Is there a national website for medical patient information, set up/approved by the MoH or a FP professional association? | Y/N  Weblink: | Source 1: internet (for 11 countries:  Source 2: National expert / NPM |

| **DOMAIN 2. Systemic Aspects of Facility Management** (7 indicators) | | | |  |
| --- | --- | --- | --- | --- |
| DIMENSION 2.1 Scale of PC Delivery (1 indicator) | | Coding / answering categories | Source(s) | |
| 2.1.1 | Which percentage of FPs are working in the following practice settings?  *(NB: other PC disciplines: e.g. physiotherapist; social worker; speech therapist)* | - 1 FP (with or without a nurse): …… % - 2 or more FPs (with or without a nurse): …… % - 1 or 2 FPs (with or without a nurse) plus other PC discipline(s): …… % - 3 or more FPs (with or without a nurse) plus PC discipline(s) …… % - FPs in other settings, namely ……. …. % | Source: National expert / NPM | |
| DIMENSION 2.2 Systems/ structures for Quality Assurance and Safety  (4 indicators) | | Coding / answering categories | Source(s) | |
| 2.2.1 | Do the following mechanisms exist for FPs and PC facilities to operate?   - Licensure (or: registration) = legal mandatory permission for *individual* FPs to practice - Re-licensure = mandatory periodical update of the *individual* FP license - Certification of additional qualifications = *voluntary* independent assessment of *individual* providers on competences - Practice certification = *voluntary* assessment of PC *facilities* or practices on organisational aspects (e.g. safety) | - Licensure (registration)   - Yes   - No - Re-licensure   - Yes   - No - Voluntary individual certification of qualifications   - Yes   - No - Voluntary practice certification   - Yes   - No | Source 1: EURACT CME/CPD database (re: re-licensure)  (39 European countries) <https://www.euract.eu/country-database-entries/index/93c7b61e-25f3-4dde-89b6-2ce13168484a>  Source 2: National expert / NPM | |
| 2.2.2 | Is the basis for re-licensure as a FP the number of CME points obtained? | - Yes - No | Source 1: EURACT CME/CPD database (account needed) (39 European countries) <https://www.euract.eu/country-database-entries/index/93c7b61e-25f3-4dde-89b6-2ce13168484a>  Source 2: National expert / NPM | |
| 2.2.3 | Are community health surveys conducted to improve the quality and responsiveness of PC? | No  Yes, at the following scale:   - Nationwide Yes/No - At local / regional level Yes/No | Source: National expert / NPM | |
| 2.2.4 | Are patient experiences measured at facility level? | - No (or very rarely) - Yes, incidentally - Yes, widespread | Source: National expert / NPM | |
| DIMENSION 2.3 Practice Management Incentives  (1 indicator) | | Coding / answering categories | Source(s) | |
| 2.3.1 | Is an allocated budget available for PC/FP practices to pay a (part-time) practice manager? | - Yes - No | Source: National expert / NPM | |
| DIMENSION 2.4 Community Involvement (1 indicator) | | Coding / answering categories | Source(s) | |
| 2.4.1 | To what extent do citizens/ patient representatives have any formal role in the areas specified? | Training/education for patients   - Strong - Incidental / developing - Weak/ No   Membership in PC advisory boards at community level (e.g. council boards)   - Strong - Incidental / developing - Weak/ No   Membership in supervisory boards of PC facilities   - Strong - Incidental / developing - Weak/ No | National expert / NPM | |

| **DOMAIN 3. Systemic Aspects of Care Delivery** (17 indicators) | | | |  |
| --- | --- | --- | --- | --- |
| DIMENSION 3.1 Accessibility (6 indicators) | | Coding / answering categories | Source(s) | |
| 3.1.1 | The total number of (directly accessible) active FPs available per 100,000 population | - FPs: …. per 100.000 | Source 1: (partly/t.b.s) WHO/HlthRes-DB. [European database on human and technical resources for health - European Health Information Gateway (who.int)](https://gateway.euro.who.int/en/datasets/european-database-on-human-and-technical-resources-for-health/)  Source 2: National expert / NPM | |
| 3.1.2 | Difference between region, province or state with highest and with lowest density of FPs (per 100,000 population) | FPs …. per 100.000 in highest region  FPs …. per 100.000 in lowest region | Source 1: Eurostat NUTS 2 or 3 (depending on availability)  Source 2: National expert / NPM | |
| 3.1.3 | Difference between average urban density of FPs (per 100,000 population) and average rural density of FPs | Urban average FPs per 100.000 ……..  Rural average FPs per 100.000 ….. | Source: National expert / NPM | |
| 3.1.4 | Do (regional or national) shortages exist of FPs according to usual national norms? | Nationwide Yes / No  In certain regions Yes / No | Source: National expert / NPM | |
| 3.1.5 | Are FP practices or PC centres obliged to have a minimum number of opening hours or days? | Yes / No | Source: National expert / NPM | |
| 3.1.6 | How is out-of-hours PC organised? Indicate for each of the specified models to what extent it is used? | Models of out-of-hours PC:  Solo FP is available for his/her own practice only   - (Almost) always used - Usually used - Occasionally used - Seldom or never used   Group of FPs on a rota basis is available for the practices of the group   - (Almost) always used - Usually used - Occasionally used - Seldom or never used   Larger group of FPs are working in special OOH facility with support staff   - (Almost) always used - Usually used - Occasionally used - Seldom or never used   FPs are working at Emergency department of hospital   - (Almost) always used - Usually used - Occasionally used - Seldom or never used   No FP-based OOH services exist; patients attend hospital   - (Almost) always used - Usually used - Occasionally used - Seldom or never used   Commercial deputizing services are hired during OOH   - (Almost) always used - Usually used - Occasionally used - Seldom or never used   Other: ……… (please specify) | Source 1: Steeman et al, 2020  Source 2: National expert / NPM | |
| DIMENSION 3.2 Comprehensiveness  (6 indicators) | | Coding / answering categories | Source(s) | |
| 3.2.1 | In which organisational way are the specified screening programmes delivered? | Cervical cancer screening   - Integrated into PC: Y/N - In PC but organized as a vertical programme: Y/N - As a vertical programme (not PC) Y/N   Breast cancer screening   - Integrated into PC: Y/N - In PC but organized as a vertical programme: Y/N - As a vertical programme (not PC) Y/N   Colon cancer screening   - Integrated into PC: Y/N - In PC but organized as a vertical programme: Y/N - As a vertical programme (not PC) Y/N | Source: National expert / NPM | |
| 3.2.2 | Are the specified vaccination services being carried out in PC as part of a national vaccination programme? | - HPV vaccination for girls: Y/N - HPV vaccination for boys: Y/N - Influenza vaccination for at risk population: Y/N - DTP (Diphtheria, Tetanus and Pertussis) vaccination: Y/N - Measles vaccination: Y/N - Hepatitis B vaccination: Y/N - Mumps vaccination: Y/N - Rubella vaccination: Y/N | Source: National expert / NPM | |
| 3.2.3 | To what extent will patients with the specified health problems visit a FP for first contact care? | Child with severe cough   - (Almost) always - Usually - Occasionally - Seldom or never   Child aged 8 with hearing problem  o (almost) always  o Usually  o Occasionally  o Seldom or never  Woman aged 18 asking for oral contraception  o (almost) always  o Usually  o Occasionally  o Seldom or never  Woman aged 35 with irregular menstruation  o (almost) always  o Usually  o Occasionally  o Seldom or never  Woman aged 35 with psychosocial problems  o (almost) always  o Usually  o Occasionally  o Seldom or never  Woman (aged 50) with a lump in her breast  o (almost) always  o Usually  o Occasionally  o Seldom or never  Man (aged 28) with a first convulsion  o (almost) always  o Usually  o Occasionally  o Seldom or never  Man (aged 52) with alcohol addiction problems  o (almost) always  o Usually  o Occasionally  o Seldom or never | Source: National expert / NPM | |
| 3.2.4 | How is the coverage of medicines prescribed at PC level defined? | Yes   - there is a positive list of medicines (those covered) - there is a negative list (those not covered)   No (such restrictions do not exist) | Source 1: OECD HSC Survey (q.59 ‘medicines’)  Source 2: National expert / NPM | |
| 3.2.5 | To what extent do FPs / PC practices provide the following health services to their patients who need so? | Family planning /contraceptive care  o (almost) always  o Usually  o Occasionally  o Seldom or never  Routine antenatal care (in the context of a national scheme)  o (almost) always  o Usually  o Occasionally  o Seldom or never  Routine pediatric surveillance to children (up to 4 years)  o (almost) always  o Usually  o Occasionally  o Seldom or never  Palliative care  o (almost) always  o Usually  o Occasionally  o Seldom or never | Source: National expert / NPM | |
| 3.2.6 | To what extent is social prescribing in PC practices recognised?  *(Social prescribing means that FPs can refer patients to non-clinical social programmes in the community. The focus can be on i.a. income, health food, sports, housing, social activation, informal care support)* | - Social prescribing is formally recognized and increasingly practiced - Social prescribing is being discussed but not (or hardly) practiced - Social prescribing is currently (practically) unknown | Source 1: Literature; answers for 12 countries  Source 2: National expert / NPM (for other countries) | |
| DIMENSION 3.3 Continuity (3 indicators) | | Coding / answering categories | Source(s) | |
| 3.3.1 | To what extent are patients generally free to choose a FP or PC practice? (*choose most applicable option)* | - The patient is assigned to a specific provider (e.g. a health centre serving a geographical area) Y/N - The patient’s choice is limited (e.g. to a small geographical area or a specific network of providers) Y/N - Patients are not obliged to register with a PC practice/FP but there are (financial) incentives (e.g. reduced co-payments) to do so Y/N - No such incentive, encouragement or obligation to register Y/N | Source 1: OECD/HSC Survey (q.39a)  Source 2: National expert / NPM | |
| 3.3.2 | Can patients choose his/her individual care provider (FP) within the PC practice chosen or assigned to? | - Yes, patients can freely choose FP Y/N - No, patients cannot choose FP Y/N - Not relevant (PC services are predominantly provided in solo practices) Y/N | Source 1: HiT (Ch5)  Source 2: National expert / NPM | |
| 3.3.3 | Are people registered with a FP/ or PC practice? | Yes   - almost) the whole population (>95%) Y/N - the majority (>50%) Y/N - less than 50% Y/N   No | Source 1: OECD/HSC Survey (q.37)  Source 2: HiT (Ch5)  Source 3: National expert / NPM | |
| DIMENSION 3.4 Coordination (2 indicators) | | Coding / answering categories | Source(s) | |
| 3.4.1 | To what extent do FPs control access to (medical) specialist care? | - FP referral is compulsory to access most types of (medical) specialist care (except in case of emergency) Y/N - FP referral is compulsory to access restricted types of (medical) specialist care (except in case of emergency) Y/N - Patients have financial incentives to obtain a FP’s referral (e.g. reduced co-payments), but direct access is always possible Y/N - There is no need and no incentive to obtain FP referral Y/N | Source 1: OECD/HSC Survey (q.38)  Source 2: HiT  Source 3: National expert / NPM | |
| 3.4.2 | To what extent do FPs control access to specialised mental health care? | - FP referral is compulsory to access specialised mental health services Y/N - Referral is not compulsory but patients have financial incentives to obtain a FP referral (e.g. reduced co-payments) Y/N - There is no need and no incentive to obtain a FP referral Y/N | Source 1: HiT (section 5.10 or 5.11). Results extracted for 18 countries  Source 2: National expert / NPM | |
